# Comparative Study for the Presence of Enterococcal Virulence Factors Gelatinase, Hemolysin and Biofilm Among Clinical and Commensal Isolates of *Enterococcus Faecalis*

**DOI:** 10.4103/0974-2727.72159

**Published:** 2010

**Authors:** P M Giridhara Upadhyaya, B L Umapathy, K L Ravikumar

**Affiliations:** Department of Microbiology, Kempegowda Institute of Medical Sciences, Bangalore, Karnataka, India; 1 Department of Microbiology, PGI-ESIMSR, Model Hospital, Bangalore, Karnataka, India

**Keywords:** Biofilm, Enterococcus, gelatinase, hemolysin, virulence factors

## Abstract

**Background:**

Biofilm production, gelatinase and hemolysin are the potential virulence factors of Enterococci. Gelatinase and hemolysin producing strains of *Enterococcus Faecalis* have been shown to cause severe infections in animal models. Biofilm production has been shown to enhance the persistence of *E. faecalis* in urinary bladder and other medical indwelling devices infections.

**Aims:**

To compare the presence of gelatinase, hemolysin and biofilm formation among clinical and commensal isolates and to study the co-relation between virulence factors with respect to different clinical specimens.

**Settings and Design:**

During the study period of 2 years from July 2004 to July 2006, 200 clinical isolates from nosocomial infections and 100 commensal isolates of *E. faecalis* were taken for the study.

**Materials and Methods:**

The clinical and commensal isolates were tested for the presence of gelatinase, hemolysin and biofilm and compared. The presence of these virulence factors among different clinical isolates was also studied.

**Statistical Analysis:**

Chi-square and likelihood ratio analysis were carried out using SSPS version 5.1 software.

**Results:**

The clinical isolates produced 39, 16.5 and 32.5% of gelatinase, hemolysin and biofilm, respectively, as compared to 31, 19 and 16% produced by the commensal isolates, respectively. Endotracheal tube infection, urinary tract infection, umbilical catheter tip infected isolates produced 60.8, 86.6 and 100% biofilm, respectively.

**Conclusion:**

Significant difference in the production of biofilm (*P*<0.001) was noted between clinical and commensal isolates. Organism isolated from medically indwelling devices produced high amount of biofilm, confirming its role in colonization and causing nosocomial infections.

## INTRODUCTION

Enterococci are gram positive bacteria that normally inhabit the gastrointestinal tract of many animals including humans. However, when they colonize habitats where they are not normally found, these opportunistic bacteria can become pathogens.[1]

Incidence of Enterococcal infections, especially hospital acquired, has dramatically increased over the last 25 years. Enterococci have been reported recently as a major cause of nosocomial infections, being increasingly detected in postoperative wound infections, blood stream and urinary tract infections (UTIs). *Enterococcus Faecalis* is responsible for approximately 80–90% of all Enterococcal infections.

They are intrinsically resistant to or tolerant to many antibiotics and are readily able to acquire resistance to antibiotics, either by mutation or by acquisition of plasmids or transposons containing genetic sequences that confer resistance in other bacteria.[2] A number of studies have identified different virulence factors, the most important among them being[3–9] gelatinase, hemolysin, enterococcal surface protein (Esp), aggregation substance (AS), MSCRAMM Ace (microbial surface component recognizing adhesive matrix molecule adhesion of collagen from Enterococci), serine protease, capsule, cell wall polysaccharide and superoxide. These factors have been associated with the virulence of *E. faecalis* in animal models.[10–13] It is not clear whether the presence of these factors in *E. faecalis* isolates from clinical and commensal isolates contribute to the virulence in humans.

Esp or biofilm is a cell wall associated protein in *E. faecalis* isolates. Frequency of gene coding for Esp has been higher among clinical isolates than among commensal isolates.[14] Esp is shown to enhance the persistence of *E. faecalis* in urinary bladder during experimental UTIs.

Gelatinase is a protease produced by *E. faecalis*. It is capable of hydrolyzing collagen, casein, hemoglobin and other peptides.[10] Gelatinase producing strains of *E. faecalis* have been shown to contribute to the virulence of endocarditis in an animal model. Hemolysin is a cytolytic protein capable of lysing human, horse and rabbit erythrocytes. Hemolysin producing strains are found to be associated with increased severity of infections.[15]

The present study evaluates *E. faecalis* isolates from nosocomial infections and stool samples to compare the production of these three virulence factors between clinical and commensal isolates.

## MATERIALS AND METHODS

Three hundred and ninety-seven *Enterococcus* spp. were isolated over a period of 2 years from different clinical specimens. Two hundred isolates were confirmed as *E. faecalis* by biochemical reactions[16] and taken up for the study. Hundred commensal isolates of *E. faecalis* were isolated from stool samples. Isolates were grown on trypticase-soy agar for subsequent testing.

**Figure 1:**
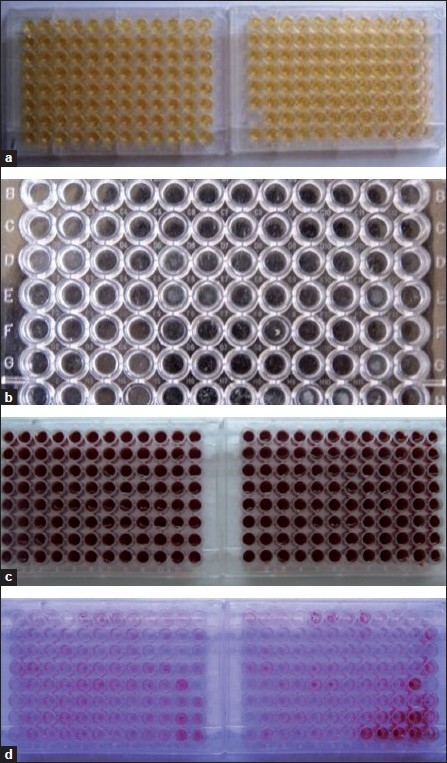
(a) *E. faecalis* isolates inoculated in microtiter plates containing TSB with 0.5% glucose; (b) plates incubated with *E. faecalis* for 48 hours washed three times with distilled water; (c) biofilm containing plates stained with 0.1% safranin for 20 minutes; (d) wells in microtiter plates showing biofilm formation after staining with safranin

Biofilm[17] formation was detected by inoculating the isolates into trypticase-soy broth [TSB] with 0.5% glucose and incubated at 37°C. After overnight incubation, the culture was diluted 1:40 in fresh TSB–0.5% glucose. Two hundred microliters of the diluted solution was added to flat-bottomed polystyrene microtiter well and incubated for 48 hours at 37°C. Wells were gently washed three times with distilled water. After drying the plates in an inverted position at room temperature for 1 hour, the adherent biofilm was stained with 0.1% safranin and allowed to stand for 20 minutes at room temperature. Absorbance of the biofilm on the bottom surface of each well of the dried plates was determined at 490 nm in an enzyme-linked immunosorbent assay (ELISA) reader. Test was carried out in triplicate and the average of the three optical density (OD) values was taken. Culture medium without organism was taken as blank. Biofilm producing *E. faecalis* OG1RF was taken as positive control. Mean OD value of positive control was taken as standard. Those values above 0.2 were considered as high biofilm producers. Values below 0.081 were categorized into low or non-biofilm producers. OD values above the standard but within 0.081 and 0.2 were taken as moderate biofilm producers [Figure 1a–d].

Hemolysin production was detected by inoculating Enterococci onto freshly prepared beef heart infusion agar supplemented with 5% horse blood. Plates were incubated overnight at 37°C in a carbon dioxide chamber and[2] evaluated at 24 and 48 hours. A clear zone of β-hemolysis around the streak on horse blood agar was considered to be a positive indication of hemolysin production. Gelatinase production was detected by inoculating the organism onto freshly prepared peptone-yeast extract agar containing 30 g/L of gelatine.[2] Plates were incubated overnight at 37°C and then cooled to ambient temperature for 2 hours. The appearance of a turbid halo or zone around the colonies was considered to be a positive indication of gelatinase production.

Testing for susceptibility to Vancomycin and Linezolid was done with the use of E test strips (Hi-Media, Mumbai, India). Minimum inhibitory concentration Multi Drug Resistant (MDR) break points recommended by Clinical and Laboratory Standards Institute (CLSI)[18] were used.

## RESULTS

### Production of biofilm, hemolysin and gelatinase

Seventy-eight (39%) clinical isolates and 31 (31%) commensal isolates were gelatinase producing and 33 (16.5%) clinical and 19 (19%) commensal isolates produced hemolysin.

Sixty-five (32.5%) clinical isolates were positive for biofilm production, out of which 23 (11.5%) were high biofilm producers and the remaining 42 (21%) were moderate biofilm producers.

Among the commensal isolates, 16 (16%) were biofilm producers with 4 producing high and 12 producing moderate amounts of biofilm [Table 1].

**Table 1:** Gelatinase, hemolysin and biofilm production between clinical and commensal isolates

Virulence factors	Clinical isolates (200)	Commensal isolates (100)
Gelatinase	78	31
Hemolysin	33	19
Biofilm	65	16

Table 2 shows the age, sex and clinical cases (no. and %) from which *E. faecalis* was isolated.

**Table 2:** Demographic and clinical characteristics of 200 patients with nosocomial infection due to *E. faecalis*

Characteristics	Value no. (%)
Age, median years (range)	16–66
Sex, no. of males/no. of females	131/69
Postoperative wound infection	125 (62.5)
UTI	15 (7.5)
Diabetic foot infection	20 (10)
Infected compound fracture	07 (3.5)
Endotracheal tube infection	23 (11.5)
Umbilical catheter tip infection	02 (1)
Burn case	03 (1.5)
Septicemia	05 (2.5)

Data are no. (%) of patients unless indicated otherwise

### Production of combination of hemolysin, gelatinase and biofilm

Eighteen (9%) clinical isolates produced all the three virulence factors. Fifteen (7.5%) isolates produced biofilm and gelatinase. Seven (3.5%) isolates produced biofilm and hemolysin. Six (3%) isolates produced gelatinase and hemolysin. Twenty-five (12.5%) isolates produced only biofilm. Thirty-nine (19.5%) and two (1%) isolates were positive for only gelatinase and hemolysin production, respectively.

Among the commensal isolates, 6 (6%) were positive for all the three factors. Two (2%) were positive for biofilm and gelatinase, and one (1%) was positive for biofilm and hemolysin production. Seven (7%) isolates produced only biofilm. Twenty-three (23%) and 12 (12%) were positive for only gelatinase and hemolysin production, respectively.

### Relationship between clinical characteristics and virulence factors

The relationship between clinical characteristics and virulence factors is shown in Tables 3 and 4.

**Table 3:** Production of the three virulence factors in isolates from different clinical conditions

Clinical samples	Gelatinase (%)	Hemolysin	Biofilm
Postoperative wound infection (*n*=125)	44 (35.2)	22 (17.6)	27 (21.6)
UTI (*n*=15)	10 (66.6)	6 (40)	13 (86.6)
Diabetic foot infection (*n*=20)	11 (55)	2 (10)	5 (25)
Infected compound fracture (*n*=07)	3 (42.8)	–	2 (28.5)
Endotracheal tube infection (*n*=23)	7 (30.4)	3 (13)	14 (60.8)
Umbilical catheter tip (*n*=02)	1 (50)	–	2 (100)
Burns case(*n*=03)	1 (33.3)	–	–
Septicemia (*n*=05)	1 (20)	–	2 (40)

**Table 4:** Production of combination of different virulence factors among clinical isolates

Clinical samples	G + H + B (%)	G + H	G + B	H + B	G	H	B
Postoperative wound infection (*n*=125)	12 (9.6)	4 (3.2)	4 (3.2)	4 (3.2)	24 (19.2)	2 (1.6)	07 (5.6)
UTI (*n*=15)	3 (20)	1 (6.6)	5 (33.3)	2 (13.2)	1 (6.6)	–	3 (20)
Diabetic foot infection (*n*=20)	1 (5)	1 (5)	2 (10)	–	7 (35)	–	2 (10)
Infected compound fracture (*n*=07)	–	–	1 (14.2)	–	2 (28.4)	–	1 (14.2)
Endotracheal tube infection (*n*=23)	2 (8.7)	–	2 (8.7)	1 (4.3)	3 (12.9)	–	9 (38.7)
Umbilical catheter tip (*n*=02)	–	–	1 (50)	–	–	–	1 (50)
Burns case (*n*=03)	–	–	–	–	1 (33.3)	–	–
Septicemia (*n*=05)	–	–	–	–	1 (20)	–	2 (40)

G + H + B = gelatinase, hemolysin and biofilm producing isolates; G + H = gelatinase and hemolysin producing isolates; G + B = gelatinase and biofilm producing isolates; H + B = hemolysin and biofilm producing isolates; G = isolates producing only gelatinase; H = isolates producing only hemolysin; B = isolates producing only biofilm

### Relationship between virulence factors and antibiotic susceptibility

Among the *E. faecalis* clinical isolates tested, one (0.5%) was resistant for Vancomycin and none of the isolates were resistant to Linezolid. All the commensal isolates were sensitive to Vancomycin and Linezolid. Clinical isolate which was resistant to Vancomycin was positive for biofilm production.

## DISCUSSION

*E. faecalis* is an important cause of hospital borne infections. We determined and compared the prevalence of three virulence factors, biofilm, hemolysin and gelatinase, among clinical and commensal isolates. Study shows that with respect to hemolysin and gelatinase production, there is no significant difference between the clinical and commensal isolates (*P* ≥ 0.174).

Nosocomial strains of organisms develop various mechanisms in colonizing to cause infection. Biofilm production is an important factor which helps the organism to adhere onto surfaces, which facilitates later in invasion and causing infection. This study shows a significant difference in the production of biofilm (*P*<0.001).

A comparative study among the different clinical isolates with respect to production of the three virulence factors shows that biofilm production is very high among isolates grown from UTI, endotracheal tube infection and umbilical catheter tip infection as shown in Table 3.

We conclude that biofilm production in nosocomial strains of organisms is an important pathogenic factor in causing infection in the hospital environment. Other virulence factors also have a role in the occurrence of infection and in the clinical outcome. Further study on the other virulence factors will throw some light on the mechanism of pathogenesis in *E. faecalis*.

There was no significant relationship between virulence factors, ability to cause infection and antibiotic susceptibility to Vancomycin and Linezolid, as only one clinical isolate was resistant to Vancomycin and none for Linezolid.

Given the importance of Enterococcus as a nosocomial pathogen and increasing prevalence of MDR Enterococcus as shown by other studies,[19] the identification of virulence factors associated with invasiveness and disease severity has become an important subject for research. Development of other mechanisms like blocking of Enterococcal biofilm production or inhibiting the action of other virulence factors may provide an alternate method of therapy in the face of antimicrobial resistance.
